# Genome Sequence of *Borrelia crocidurae* Strain 03-02, a Clinical Isolate from Senegal

**DOI:** 10.1128/genomeA.01150-14

**Published:** 2014-11-06

**Authors:** Aurélien Fotso Fotso, Oleg Mediannikov, Roshan Padmanabhan, Catherine Robert, Pierre-Edouard Fournier, Didier Raoult, Michel Drancourt

**Affiliations:** a URMITE, UMR6236, CNRS 7278, IRD 198, INSERM 1095, IFR 48, Méditerranée Infection, Faculté de Médecine, Aix-Marseille Université, Marseille, France; b URMITE, UMR, IRD 198, Campus IRD Ham Maristes, Dakar, Senegal

## Abstract

The draft genome sequence of *Borrelia crocidurae* strain 03-02, a blood isolate from a febrile Senegalese patient, comprises a 920,021-bp linear chromosome (27.7% G+C content), 32 tRNAs, 818 open reading frames, and one cluster of regularly interspaced short palindromic repeats. Its genotype differs from that of the Achema reference strain.

## GENOME ANNOUNCEMENT

*Borrelia crocidurae* is a spirochete responsible for tick-borne relapsing fever in West Africa (1). It is maintained in a triangle that involves humans, *Ornithodoros sonrai* soft ticks, and rodents (2, 3). Here, we report the draft genome of an isolate from a febrile patient with relapsing fever in rural Senegal (3). For isolation, blood was inoculated in Barbour-Stoenner-Kelly-H (BSK-H) medium (Sigma, Saint-Quentin-Fallavier, France) supplemented with 10% heat-inactivated rabbit serum (Eurobio, Courtaboeuf, France) at 32°C (4).

*B. crocidurae* strain 03-02 (deposited in the Collection de Souches de l’Unité des Rickettsies, CSUR P235) genome was sequenced by combining paired-end libraries and a bar code strategy in order to be mixed with 11 other genomic projects constructed according to the Nextera XT library kit using high-throughput MiSeq Technology (Illumina, San Diego, CA). Genomic DNA extracted using a phenol-chloroform protocol was quantified by a Qubit assay at 100 ng/µl, and 1 ng was used as input. The “tagmentation” step generated inserts in the range of 700 bp to 1 kb, validated on a Caliper Lab Chip (PerkinElmer). Lab chip PCR amplification completed the tag adapters and introduced dual-index bar codes. After purification on Ampere beads, the library was normalized on specific beads, according to the Nextera XT protocol. The pooled single-strand library was loaded onto the reagent cartridge and then onto the MiSeq instrument, along with the flow cell. Automated cluster generation and paired-end sequencing with dual-index reads was performed in a single 39-h 2 × 250 bp run. The total run information of 7.64 Gb was obtained from a 524,000/mm^2^ cluster density, with a cluster passing quality control filters of 96.10% (12,380,000 clusters). Within this run, the index representation for *B. crocidurae* strain Achema was determined to 2.97%. A total of 367,964 paired-end reads were mapped to *B. crocidurae* strain Achema (4) using the CLC Genomics Workbench software package 6.0.1 (CLC bio, Denmark) and generated one scaffold. A preliminary open reading frame (ORF) prediction was conducted by automated annotation with Prokka (http://www.vicbioinformatics.com/software.prokka.shtml) and RAST (5). Clusters of regularly interspaced short palindromic repeats (CRISPRs) were detected using the CRISPR finder (http://crispr.u-psud.fr/Server/).

The 920,021-bp linear chromosome of *B. crocidurae* strain 03-02 is 0.6% larger than that of strain Achema (919,477 bp) (4), yet it contains 818 ORFs (85.26% of the proteins it encodes are listed in the COG database) compared to 865 ORFs for strain Achema (79% of the proteins it encodes are listed in the COG database) and 32 tRNAs. Its G+C content is 27.70%. Accordingly, the nucleotide similarity at the genome level between *B. crocidurae* strains Achema and 03-02 is 98.88%. *In silico* multispacer sequence typing of *B. crocidurae* 03-02 strain found sequence type 6 (ST6), a genotype previously found in patients with relapsing fever in rural Senegal. It differed in the five spacer sequences used from the type strain Achema, isolated from an *Ornithodoros* tick from Mauritania (6). No antibiotic resistance genes were found in the genome using the ResFinder tool.

### Nucleotide sequence accession number.

The genome sequence from *B. crocidurae* strain 03-02 has been deposited in EMBL-EBI under the accession no. CCXD00000000.
